# Dynamic Changes in Serum Neutrophil Gelatinase-Associated Lipocalin for Discrimination of Creatinine-Defined Acute Kidney Injury After Laparoscopic Sleeve Gastrectomy: A Prospective Observational Cohort Study

**DOI:** 10.3390/jcm15197466

**Published:** 2026-09-25

**Authors:** Remzi Can Çakır, Zinet Asuman Arslan Onuk, Sibel Kulaksızoğlu, Halit Özgül, Arif Aslaner, Erhan Aydemir, Ömer Çelik, Rıdvan Yavuz, Azmi Lale, Orhan Aras

**Affiliations:** 1 Department of General Surgery, Bursa City Hospital, University of Health Sciences, 16110 Bursa, Turkey; 2 Department of Anesthesiology and Reanimation, Antalya Education and Research Hospital, University of Health Sciences, 07100 Antalya, Turkey; 3 Department of Biochemistry, Antalya Education and Research Hospital, University of Health Sciences, 07100 Antalya, Turkey; 4 Department of General Surgery, Antalya Education and Research Hospital, University of Health Sciences, 07100 Antalya, Turkey; 5 Department of Gastroenterological Surgery, Antalya Education and Research Hospital, University of Health Sciences, 07100 Antalya, Turkey; 6 Department of Surgical Oncology, Erciyes University, 38039 Kayseri, Turkey; dr.azmilale@gmail.com

**Keywords:** acute kidney injury, neutrophil gelatinase-associated lipocalin, NGAL, ΔNGAL, laparoscopic sleeve gastrectomy, bariatric surgery, metabolic surgery, renal biomarkers, serum creatinine

## Abstract

**Background/Objectives:** Acute kidney injury (AKI) may occur after laparoscopic sleeve gastrectomy (LSG) as a result of obesity-related renal vulnerability, pneumoperitoneum-induced changes in renal perfusion, and perioperative hemodynamic stress. Serum creatinine remains the standard diagnostic marker for AKI; however, it may not fully reflect perioperative renal stress. This study aimed to evaluate the diagnostic performance of serum neutrophil gelatinase-associated lipocalin (NGAL), particularly dynamic changes in NGAL (ΔNGAL), for discriminating between patients with and without postoperative AKI after LSG. **Methods:** This prospective observational cohort study included 60 patients with severe obesity who underwent LSG at a tertiary referral center. Serum NGAL, serum creatinine, blood urea nitrogen, and estimated glomerular filtration rate were measured preoperatively and on postoperative days 1 and 3. AKI was defined according to creatinine-based Kidney Disease: Improving Global Outcomes criteria. Patients were compared according to postoperative AKI status. ΔNGAL and changes in renal function parameters were analyzed, and receiver operating characteristic curve analysis was performed to assess diagnostic performance. **Results:** AKI developed in 6 patients (10%). Postoperative day 1 NGAL levels were significantly higher in patients who developed AKI than in those who did not. ΔNGAL from preoperative baseline to postoperative day 1 was significantly greater in the AKI group. ROC analysis demonstrated that ΔNGAL had strong discriminatory performance for postoperative AKI, with an area under the curve of 0.901, sensitivity of 83.3%, and specificity of 90.7% at an exploratory cut-off value of 57.2 ng/mL. Absolute NGAL values showed lower diagnostic performance than ΔNGAL. **Conclusions:** Dynamic changes in serum NGAL should not be interpreted as a replacement for creatinine-based AKI criteria but may be considered a complementary biomarker associated with perioperative renal stress in selected bariatric surgery patients.

## 1. Introduction

LSG is one of the most frequently performed metabolic and bariatric surgical procedures worldwide [1]. Although LSG is generally considered safe, patients with severe obesity may be vulnerable to perioperative renal stress because of obesity-related alterations in renal hemodynamics, increased inflammatory burden, and reduced renal functional reserve. In addition, procedure-related factors such as pneumoperitoneum, increased intraabdominal pressure, reverse Trendelenburg positioning, perioperative fluid shifts, and transient hemodynamic changes may contribute to postoperative renal hypoperfusion and increase the risk of AKI [2,3].

AKI after bariatric surgery is clinically relevant because even transient postoperative renal dysfunction may affect perioperative management, length of hospital stay, and long-term renal outcomes. The Kidney Disease: Improving Global Outcomes (KDIGO) criteria define AKI based on increases in serum creatinine and/or reductions in urine output [4]. However, serum creatinine is a delayed functional marker and may not rise until a measurable decline in glomerular filtration has occurred. Therefore, reliance on creatinine alone may limit the identification of renal stress, particularly in patients who develop mild or transient postoperative kidney injury.

NGAL, originally identified as a protein associated with human neutrophil gelatinase, has emerged as a promising biomarker for renal tubular injury [5]. NGAL is released from activated neutrophils and is also expressed in several tissues, including renal tubular epithelial cells, adipocytes, macrophages, and epithelial cells [6,7]. In the setting of acute renal injury, NGAL may increase earlier than serum creatinine and may therefore provide complementary information regarding renal tubular stress [8]. Nevertheless, absolute NGAL concentrations may be influenced by baseline inflammation, obesity-related metabolic activity, and nonrenal sources of NGAL expression. For this reason, the evaluation of dynamic changes in NGAL from baseline, rather than isolated single time-point measurements, may provide more clinically meaningful information in the perioperative setting.

Although previous studies have investigated NGAL in different surgical populations, data on serum NGAL dynamics after LSG remain limited. In particular, the diagnostic value of ΔNGAL for identifying postoperative renal stress in patients undergoing LSG has not been sufficiently clarified. Therefore, this prospective observational cohort study aimed to evaluate perioperative changes in serum NGAL and to compare the discriminatory performance of absolute NGAL values and ΔNGAL in relation to creatinine-defined postoperative AKI after LSG.

## 2. Materials and Methods

### 2.1. Study Design and Ethical Approval

This prospective observational cohort study was conducted at a tertiary referral center between April 2024 and September 2024. The study included patients with severe obesity who underwent elective LSG. Patients were classified into two groups according to the development of postoperative AKI: patients who developed AKI and those who did not.

The study protocol was approved by the local institutional ethics committee of Antalya Education and Research Hospital, University of Health Sciences, Antalya, Turkey (approval number: 3/1; approval date: 21 March 2024). Written informed consent was obtained from all participants. All procedures were performed in accordance with the ethical principles of the Declaration of Helsinki and its subsequent amendments.

### 2.2. Patient Selection

Patients aged 18 years or older with a body mass index (BMI) ≥ 40 kg/m^2^ who were scheduled for LSG were considered eligible for the study. Patients classified as American Society of Anesthesiologists (ASA) physical status I-III were included. Only patients without pre-existing chronic kidney disease and with available perioperative renal function and NGAL measurements were enrolled.

Patients were excluded if they had chronic kidney disease, heart failure, diabetes mellitus, hypertension, active infection, respiratory failure, or any other significant systemic disease that could independently affect renal function or NGAL levels. Patients who refused to participate and those with incomplete perioperative blood sampling were also excluded.

A total of 80 patients were assessed for eligibility. Twelve patients were excluded before analysis, and eight additional patients were excluded because of blood sampling errors. Finally, 60 patients were included in the final analysis. No formal a priori sample-size or power calculation was performed. The sample size was determined by the available NGAL ELISA capacity and study resources, allowing the inclusion of 60 patients. The patient enrollment process is summarized in Figure 1.

### 2.3. Data Collection

Demographic and clinical variables, including age, sex, height, weight, BMI, and ASA physical status, were recorded. Perioperative variables included duration of operation, duration of anesthesia, estimated intraoperative blood loss, intraoperative fluid administration, postoperative urine volume, and length of hospital stay.

Laboratory parameters were measured preoperatively and on postoperative days 1 and 3. These included serum creatinine, blood urea nitrogen (BUN), estimated glomerular filtration rate (eGFR), and NGAL. eGFR was calculated using the Chronic Kidney Disease Epidemiology Collaboration equation and expressed as mL/min/1.73 m^2^.

Inflammatory, hematological, metabolic, and nutritional parameters were also recorded, including white blood cell count, neutrophil count, lymphocyte count, neutrophil-to-lymphocyte ratio, hemoglobin, C-reactive protein, glucose, albumin, and total protein. These variables were analyzed in relation to the development of postoperative AKI.

### 2.4. Preoperative Care

All patients underwent routine preoperative evaluation, including electrocardiography, chest radiography, and standard laboratory testing. A clear liquid diet was initiated on the day before surgery, and patients were instructed to fast from midnight before the operation. Preoperative antacid therapy and antibiotic prophylaxis were administered in accordance with institutional protocols.

### 2.5. Anesthesia Technique

After arrival in the operating room, a 16-gauge peripheral intravenous catheter was inserted. Standard monitoring included five lead electrocardiography, pulse oximetry, invasive arterial blood pressure monitoring through a 20 gauge radial artery catheter, end tidal carbon dioxide monitoring, and core temperature monitoring.

Premedication was administered with intravenous midazolam at a dose of 0.04 mg/kg. Anesthesia was induced using fentanyl 2 µg/kg, propofol 1.5 mg/kg, and rocuronium 0.6 mg/kg. Anesthesia was maintained with 50% oxygen in air and desflurane at an age-adjusted minimum alveolar concentration of 0.8–1.5.

Mechanical ventilation was performed in volume-controlled mode with a tidal volume of 6–8 mL/kg, an inspiratory-to-expiratory ratio of 1:2, and a positive end-expiratory pressure of 4–7 cm H_2_O. Ventilatory settings were adjusted to maintain end-tidal carbon dioxide between 30 and 35 mmHg after establishment of pneumoperitoneum.

Intraoperative fluid management consisted of Hartmann’s solution, and systolic blood pressure was maintained at ≥90 mmHg throughout the procedure. Analgesia was provided with intravenous remifentanil infusion, and additional rocuronium was administered when required. At the end of surgery, all patients received 1000 mg intravenous paracetamol and 100 mg tramadol for postoperative analgesia. Neuromuscular blockade was reversed with neostigmine and atropine.

### 2.6. Surgical Technique

All procedures were performed laparoscopically using a standardized LSG technique. After trocar placement, carbon dioxide pneumoperitoneum was established, and intra-abdominal pressure was maintained between 12 and 14 mmHg throughout the procedure. Patients were positioned in the reverse Trendelenburg position.

The greater curvature of the stomach was mobilized, and dissection was continued up to the angle of His with identification of the crura. Gastric transection was started approximately 2–6 cm proximal to the pylorus, according to the intraoperative anatomy and surgeon’s preference. Care was taken to avoid twisting or narrowing of the gastric sleeve during stapling. An intraoperative leak test was performed at the completion of the procedure.

### 2.7. Definition of Acute Kidney Injury

Postoperative AKI was defined according to KDIGO criteria as an increase in serum creatinine of ≥0.3 mg/dL within 48 h or an increase to ≥1.5 times the baseline value [4]. Serum creatinine was measured preoperatively and on postoperative days 1 and 3. The diagnosis of AKI was primarily based on creatinine changes observed during the first 48 postoperative hours.

Postoperative urine output was measured hourly for each patient and expressed in mL/kg/h. No patient was observed to meet the KDIGO urine output criterion of <0.5 mL/kg/h for at least 6 h during postoperative monitoring; therefore, all identified AKI cases met the serum creatinine criteria. For descriptive reporting, each patient’s mean hourly urine output was calculated by summing the hourly measurements over each 24 h period and dividing by 24.

### 2.8. NGAL Measurement

Venous blood samples were obtained preoperatively and on postoperative days 1 and 3. At each predefined perioperative time point, blood samples for NGAL measurement and serum creatinine analysis were collected concurrently. Five milliliters of blood were collected into tubes containing a spray-dried clot activator and gel separator. Samples were allowed to clot at room temperature for at least 30 min and were subsequently centrifuged at 2500 rpm for 15 min at 4 °C. The separated serum samples were aliquoted into labeled cryovials, stored at −20 °C until analysis, and analyzed together in a single analytical batch after completion of sample collection.

Serum NGAL concentrations were measured using a commercially available sandwich enzyme-linked immunosorbent assay (ELISA) kit (Bioassay Technology Laboratory, Shanghai, China; Cat. No. E1719Hu) according to the manufacturer’s instructions. Each serum sample was analyzed once as a single determination; duplicate measurements were not performed. All samples were analyzed under standardized laboratory conditions after completion of sample collection. According to the manufacturer, the assay had a coefficient of variation of <10%, analytical sensitivity of 6.5 ng/mL, and measurement range of 7.81–500 ng/mL. NGAL values were expressed as ng/mL. To prevent potential assessment bias, laboratory personnel conducted all analyses completely blinded to the patients’ clinical data, postoperative outcomes, and AKI diagnostic groups.

### 2.9. Definitions of Dynamic Biomarker Changes

Dynamic changes in biomarkers were calculated as delta values between predefined time points. ΔNGAL, Δcreatinine, ΔBUN, and ΔeGFR were calculated for the following intervals: postoperative day 1 minus the preoperative value, postoperative day 3 minus the preoperative value, and postoperative day 3 minus the postoperative day 1 value. These dynamic changes were compared between patients with and without AKI.

### 2.10. Use of Generative Artificial Intelligence

During manuscript preparation, the authors used ChatGPT (GPT-5.6 Sol; OpenAI, San Francisco, CA, USA) to assist with English language editing, grammar, and readability. All AI-assisted revisions were reviewed and verified by the authors. ChatGPT was not used for data collection, statistical analysis, or interpretation of the study findings. The authors take full responsibility for the accuracy and integrity of the manuscript.

### 2.11. Statistical Analysis

Statistical analyses were performed using IBM SPSS Statistics, version 27.0 (IBM Corp., Armonk, NY, USA). The distribution of continuous variables was assessed using the one-sample Kolmogorov–Smirnov test with Lilliefors correction. Normally distributed continuous variables are presented as mean ± standard deviation, non-normally distributed continuous variables as median and interquartile range [median (Q1–Q3)], and categorical variables as number and percentage [*n* (%)].

Normally distributed continuous variables were compared between patients with and without AKI using the independent-samples *t*-test; Welch’s correction was used when the homogeneity-of-variance assumption was not met. Non-normally distributed continuous variables were compared using the two-sided Mann–Whitney U test. Categorical variables were compared using Fisher’s exact test when expected cell counts were sparse.

Correlations between changes in NGAL and changes in renal function parameters, as well as associations of preoperative NGAL with BMI, preoperative CRP, and preoperative neutrophil-to-lymphocyte ratio (NLR), were evaluated using Spearman’s rank correlation coefficient. Receiver operating characteristic (ROC) curve analysis was used to assess the ability of NGAL, ΔNGAL, Δcreatinine, and ΔBUN to discriminate patients with postoperative AKI. The area under the curve (AUC) with 95% confidence interval (CI) was calculated. Optimal cut-off values were selected using the Youden index, and sensitivity and specificity were reported with exact binomial 95% CIs. Because only six AKI events occurred, cut-off estimates were considered exploratory. A post hoc power analysis was performed for the ΔNGAL ROC curve using the Hanley–McNeil method, with a two-sided alpha level of 0.05. A two-sided *p* value < 0.05 was considered statistically significant.

Because AKI was defined using creatinine-based KDIGO criteria, the diagnostic performance of Δcreatinine was interpreted as a reference criterion rather than as an independent biomarker.

## 3. Results

A total of 60 patients who underwent LSG were included in the final analysis. The mean age was 37.8 ± 11.4 years, and 50 patients were female (83.3%). The median BMI was 44.2 (Q1–Q3: 42.0–47.9) kg/m^2^. Postoperative AKI developed in 6 patients (10.0%), whereas 54 patients did not develop AKI. All six patients with AKI met the absolute serum creatinine increase criterion of ≥0.3 mg/dL. Of these, four also met the criterion of an increase to ≥1.5 times the baseline, whereas two met only the absolute increase criterion.

There were no statistically significant differences between the AKI and non-AKI groups in age, sex, BMI, ASA physical status, duration of operation, duration of anesthesia, intraoperative blood loss, intraoperative fluid administration, or length of hospital stay. Day 1 urine output was lower in the AKI group, whereas day 3 urine output did not differ significantly. Inflammatory, hematological, metabolic, and nutritional parameters, including white blood cell count, neutrophil count, lymphocyte count, neutrophil-to-lymphocyte ratio, hemoglobin, C-reactive protein, glucose, albumin, and total protein, were comparable between the groups (Table 1).

Preoperative serum NGAL levels were similar between patients who developed AKI and those who did not. However, serum NGAL levels on postoperative day 1 were significantly higher in the AKI group than in the non-AKI group. By postoperative day 3, serum NGAL levels remained numerically higher in the AKI group, although this difference was not statistically significant.

Serum creatinine differed significantly between groups preoperatively and on postoperative day 1, but not on day 3. Preoperative eGFR was higher in the AKI group (*p* = 0.002), whereas day 1 eGFR was lower (*p* = 0.014), and day 3 eGFR did not differ significantly (*p* = 0.417). BUN did not differ between groups at any evaluated time point (Table 1).

Dynamic changes in serum NGAL were evaluated using delta values between predefined time points. ΔNGAL from baseline to postoperative day 1 was significantly greater in patients who developed AKI than in those who did not. Similarly, ΔNGAL from baseline to postoperative day 3 was significantly higher in the AKI group. In contrast, ΔNGAL from postoperative day 1 to postoperative day 3 did not differ significantly between the groups.

Changes in creatinine differed significantly between the AKI and non-AKI groups, which was as expected because AKI was defined using creatinine-based KDIGO criteria. The day 1 preoperative change in BUN showed a small between-group difference, whereas the other BUN changes were not significant. All evaluated eGFR changes differed significantly between groups (Table 2).

Spearman correlation analysis showed that ΔNGAL from baseline to postoperative day 1 was weakly positively correlated with the corresponding changes in creatinine (r_s_ = 0.297, *p* = 0.021) and BUN (r_s_ = 0.264, *p* = 0.041), and weakly negatively correlated with the corresponding change in eGFR (r_s_ = −0.339, *p* = 0.008). No other significant correlations were observed between ΔNGAL and changes in creatinine, BUN, or eGFR across the evaluated intervals (Table 3). Preoperative NGAL was not significantly correlated with preoperative CRP (r_s_ = 0.132, *p* = 0.315), BMI (r_s_ = −0.161, *p* = 0.219), or preoperative NLR (r_s_ = −0.173, *p* = 0.185).

ROC curve analysis was performed to evaluate discrimination of postoperative AKI. ΔNGAL from baseline to postoperative day 1 had an AUC of 0.901 (95% CI: 0.793–1.000). At the exploratory cut-off of 57.2 ng/mL, sensitivity was 83.3% (95% CI: 35.9–99.6%) and specificity was 90.7% (95% CI: 79.7–96.9%). The wide sensitivity interval reflects the small number of AKI events and limits the precision of the sensitivity estimate at this threshold. In the post hoc power analysis of the ΔNGAL ROC curve (α = 0.05; 6 AKI and 54 non-AKI patients), the achieved statistical power for an AUC of 0.901 was 96.5% based on the Hanley–McNeil method. The current sample size provided 80% power to detect an AUC of ≥0.834. Absolute day 1 NGAL and ΔBUN had numerically lower AUCs than ΔNGAL. Although Δcreatinine showed perfect discrimination, this result is circular and was retained only as a reference because AKI was defined according to creatinine-based KDIGO criteria. All cut-offs are exploratory and require external validation in larger cohorts (Table 4 and Figure 2).

## 4. Discussion

In this prospective observational cohort study, we evaluated perioperative changes in serum NGAL and its diagnostic performance for the identification of AKI after LSG. The main findings were as follows: first, postoperative AKI developed in 10% of patients undergoing LSG; second, serum NGAL levels on postoperative day 1 were significantly higher in patients who developed AKI; third, dynamic changes in NGAL, particularly ΔNGAL from baseline to postoperative day 1, showed stronger discriminatory performance than absolute NGAL values; and fourth, ΔNGAL demonstrated good diagnostic accuracy for AKI discrimination, with high sensitivity and specificity. These findings suggest that dynamic NGAL assessment may provide clinically useful information regarding postoperative renal stress in selected patients undergoing bariatric surgery.

Patients with severe obesity may be susceptible to perioperative renal stress because of altered renal hemodynamics, increased metabolic demand, chronic low-grade inflammation, and reduced renal functional reserve [9,10]. In addition to patient-related risk factors, LSG itself may contribute to transient renal hypoperfusion through pneumoperitoneum-induced increases in intra-abdominal pressure, reverse Trendelenburg positioning, fluid shifts, and perioperative hemodynamic changes [2,11]. Although the incidence of clinically evident AKI after bariatric surgery is generally low, even mild or transient postoperative renal dysfunction may be clinically relevant, particularly in patients with limited renal reserve or additional comorbidities [12,13,14].

Serum creatinine remains the most widely used marker for AKI diagnosis and is incorporated into the KDIGO criteria [4]. However, creatinine is a relatively inexpensive functional marker and may rise only after a measurable decline in glomerular filtration has occurred. This delayed response may limit its utility for identifying renal tubular stress. In contrast, NGAL is considered a marker of tubular injury and has been reported to increase earlier in the course of renal insult [8,15,16,17]. Therefore, NGAL may provide complementary information in the postoperative period, particularly before persistent or clinically relevant functional deterioration becomes evident.

In the present study, absolute serum NGAL levels were comparable between the groups before surgery, whereas postoperative day 1 NGAL levels were significantly higher in patients who developed AKI. This finding supports the concept that NGAL may reflect perioperative renal stress after LSG. However, absolute NGAL concentrations may be influenced by several nonrenal factors, including systemic inflammation, obesity-related metabolic activity, and perioperative stress [6,7]. Therefore, single time-point NGAL measurements may have limited specificity in surgical patients. In this context, our finding that ΔNGAL demonstrated stronger diagnostic performance than absolute NGAL is clinically important. Evaluating the individual change from baseline may reduce the effect of interindividual variability and may better reflect the acute perioperative renal response.

Because NGAL is expressed in adipocytes and may be influenced by systemic inflammation, we specifically examined whether preoperative NGAL levels were associated with BMI, preoperative CRP, or preoperative NLR. None of these correlations reached statistical significance. These findings do not support a detectable monotonic association between baseline NGAL and the selected obesity-related inflammatory markers in this cohort. Nevertheless, the absence of significant correlations does not eliminate potential obesity-related or inflammatory confounding, particularly given the modest sample size and restricted BMI range of this severely obese cohort.

Studies evaluating the relationship between NGAL, obesity, and inflammation have yielded conflicting results. Although Wang et al. reported an association between NGAL, obesity, and inflammatory pathways [18], Hardyansa et al. found no significant correlation between NGAL and BMI in non-diabetic adults [19]. Similarly, in our study, baseline serum NGAL levels showed no significant correlation with CRP, NLR, or BMI. Nevertheless, due to the small sample size and the exclusive inclusion of patients with severe obesity, a potential contribution of obesity-related systemic inflammation cannot be entirely ruled out.

The diagnostic performance of ΔNGAL from baseline to postoperative day 1 was strong in this cohort. At the optimal cut-off value, ΔNGAL showed high sensitivity and specificity for discriminating AKI. Nevertheless, these findings should be interpreted carefully. AKI was defined using creatinine-based KDIGO criteria; therefore, the excellent performance of Δcreatinine in ROC analysis was expected and should not be interpreted as an independent biomarker result. The clinically relevant interpretation is not that ΔNGAL replaces creatinine, but rather that ΔNGAL may provide additional information regarding tubular stress in patients who are at risk of AKI. Accordingly, ΔNGAL may be considered a complementary biomarker rather than an alternative diagnostic criterion.

The correlation analysis supports a cautious interpretation. ΔNGAL from baseline to postoperative day 1 showed weak positive correlations with the corresponding changes in serum creatinine and BUN, and a weak negative correlation with the corresponding change in eGFR. This direction of association is consistent with increased NGAL accompanying early functional renal impairment; however, both the small sample size and the modest strength of the correlation suggest that these findings should be considered hypothesis-generating. Larger cohorts are required to clarify the temporal and biological relationships between NGAL and conventional renal function markers.

Several studies have demonstrated that NGAL is a useful biomarker for the identification of AKI in different clinical and surgical settings. In bariatric surgery, Sidoti et al. reported that perioperative changes in NGAL and cystatin C may contribute to the early identification of renal injury [20]. Bennett et al. reported high diagnostic performance of urinary NGAL measured early after cardiac surgery, supporting its role as an early marker of postoperative renal injury [16]. Similarly, Haase et al. showed, in a multicenter pooled analysis, that NGAL positive patients, even in the absence of concomitant creatinine elevation, had worse clinical outcomes, suggesting that NGAL may identify subclinical AKI [21]. In robotic and laparoscopic surgical settings, Karaveli et al. reported that serum NGAL may be useful for detecting postoperative renal injury after robot-assisted laparoscopic prostatectomy, although specificity was limited at the proposed cut-off value [22]. In the present study, ΔNGAL demonstrated strong diagnostic performance, with higher specificity than the absolute NGAL values. These findings suggest that dynamic assessment of NGAL may provide more clinically meaningful information than isolated single time-point measurements.

From a clinical perspective, routine NGAL testing may not be necessary for all uncomplicated LSG patients. However, NGAL measurement may be useful in selected patients with increased perioperative renal risk, borderline renal function, hemodynamic instability, prolonged operative time, high intraabdominal pressure exposure, inadequate perioperative hydration, or other factors that may increase susceptibility to AKI. In such patients, identification of renal stress may allow closer monitoring, optimization of fluid therapy, avoidance of nephrotoxic agents, and individualized postoperative follow-up. Therefore, the potential value of NGAL lies not in replacing standard renal function tests, but in improving risk stratification and clinical awareness.

This study has several limitations. First, it was conducted at a single center and included a relatively small sample size. Second, the number of AKI events was low, which limits the statistical power of subgroup analyses and prevents robust multivariable modeling. The sample size was constrained by the available assay capacity, and only six patients developed AKI, limiting the precision of the diagnostic performance estimates, particularly sensitivity and the proposed cut-off. Because the ΔNGAL threshold of 57.2 ng/mL was derived and evaluated in the same cohort, its apparent performance may be optimistic. This threshold should therefore be considered exploratory and requires external validation in larger independent cohorts before clinical application. Third, all AKI cases were identified using serum creatinine criteria, as no patient met the KDIGO urine output criterion during hourly postoperative monitoring. Consequently, the findings do not establish the performance of ΔNGAL in patients with AKI defined solely by oliguria. Baseline serum creatinine was lower in the AKI group than in the non-AKI group (0.60 versus 0.83 mg/dL). This difference may reflect baseline patient characteristics or biological and analytical variability, which could not be fully disentangled in this study. Because AKI classification relied on changes in serum creatinine, this baseline imbalance may have influenced classification and between-group comparisons and should be considered when interpreting the findings. Detailed perioperative data on vasopressor use, mean arterial pressure trajectories, and net fluid balance were not available for analysis. Therefore, we could not assess between-group differences in these variables or their associations with ΔNGAL. Residual confounding by perioperative hemodynamic and fluid-related factors cannot be excluded, and the observed NGAL changes cannot be specifically attributed to pneumoperitoneum-induced renal hypoperfusion. Fourth, patients with diabetes mellitus, hypertension, chronic kidney disease, heart failure, active infection, and other major systemic diseases were excluded. Although this approach reduced confounding, it also limits the generalizability of the findings to broader bariatric populations, in whom these comorbidities are common. Fifth, NGAL is not a kidney-specific biomarker and may be influenced by inflammation, adipose tissue activity, infection, and other nonrenal sources. Although patients with overt infection or early postoperative leak were not observed in this cohort, residual inflammatory effects cannot be completely excluded. Sixth, NGAL measurements were performed as single determinations without duplicate analysis; therefore, analytical variability could not be independently assessed within this cohort. Although the post hoc analysis indicated high statistical power for testing the observed AUC against the null value, this should not be interpreted as evidence of precise estimation of the exploratory cut-off, sensitivity, or specificity, particularly given the small number of AKI events.

Despite these limitations, the prospective design, standardized perioperative sampling, and evaluation of dynamic NGAL changes strengthen the clinical relevance of the findings. The results suggest that ΔNGAL may be more informative than isolated absolute NGAL values for identifying perioperative renal stress after LSG. Larger studies, including higher-risk bariatric patients and standardized perioperative renal monitoring, are needed to determine whether NGAL-guided surveillance can improve postoperative outcomes.

## 5. Conclusions

This prospective observational cohort study demonstrated that serum NGAL, particularly its dynamic change from baseline to postoperative day 1, may provide clinically useful information for the identification of postoperative renal stress after LSG. ΔNGAL showed stronger discriminatory performance than absolute NGAL values and was associated with creatinine-based AKI development. However, ΔNGAL should not be interpreted as a replacement for conventional KDIGO criteria or serum creatinine. Instead, it may serve as a complementary biomarker for risk stratification in selected bariatric surgery patients. Larger multicenter studies, including higher-risk populations, are required to validate the proposed cut-off values and to determine whether NGAL-guided postoperative monitoring can improve clinical outcomes.

## Figures and Tables

**Figure 1:**
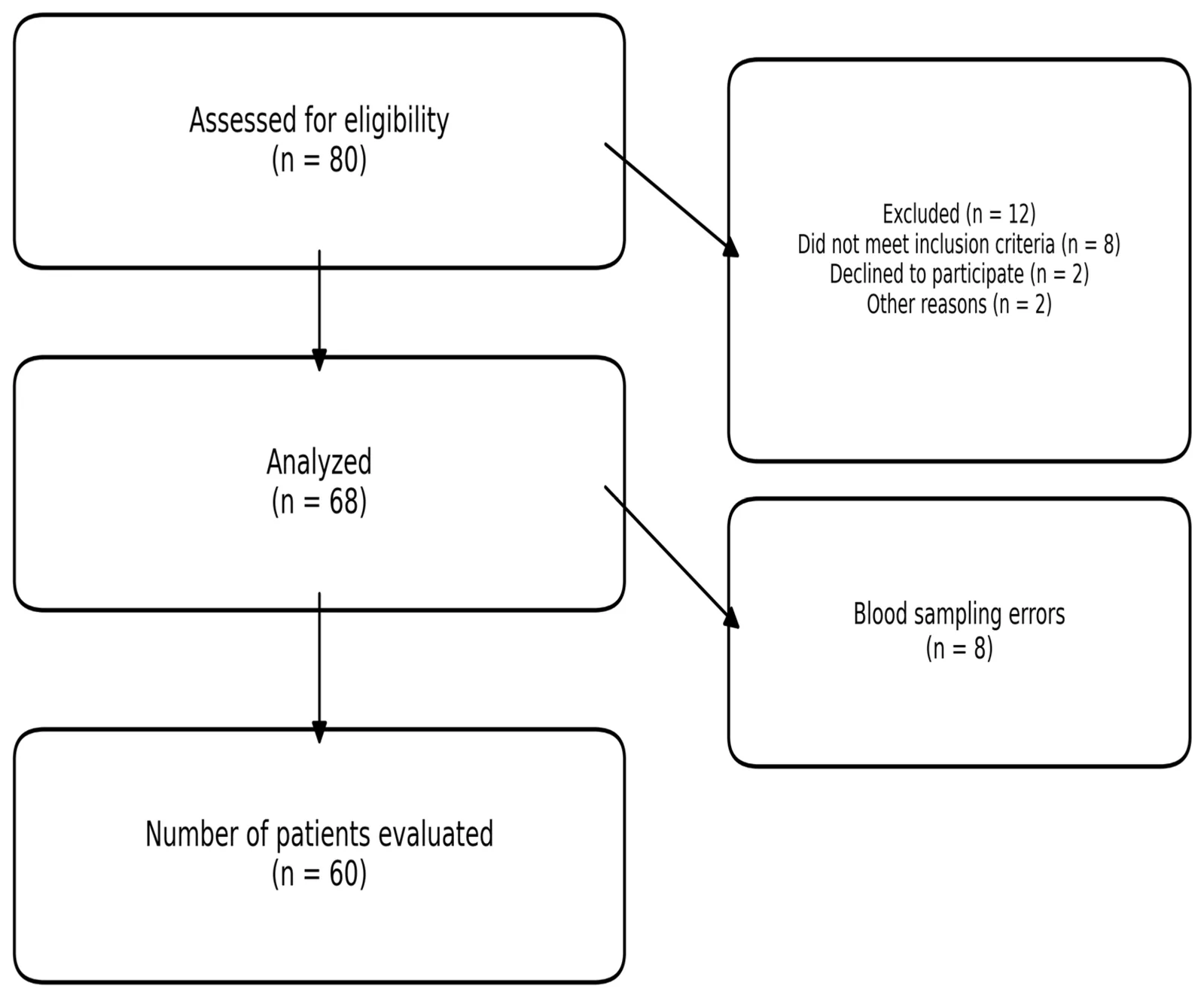
Study flow diagram. Flow diagram of patient enrollment, exclusion, blood sampling errors, and final analysis cohort. A total of 80 patients were assessed for eligibility. Twelve patients were excluded before analysis, and eight additional patients were excluded because of blood sampling errors. Finally, 60 patients were included in the study.

**Figure 2:**
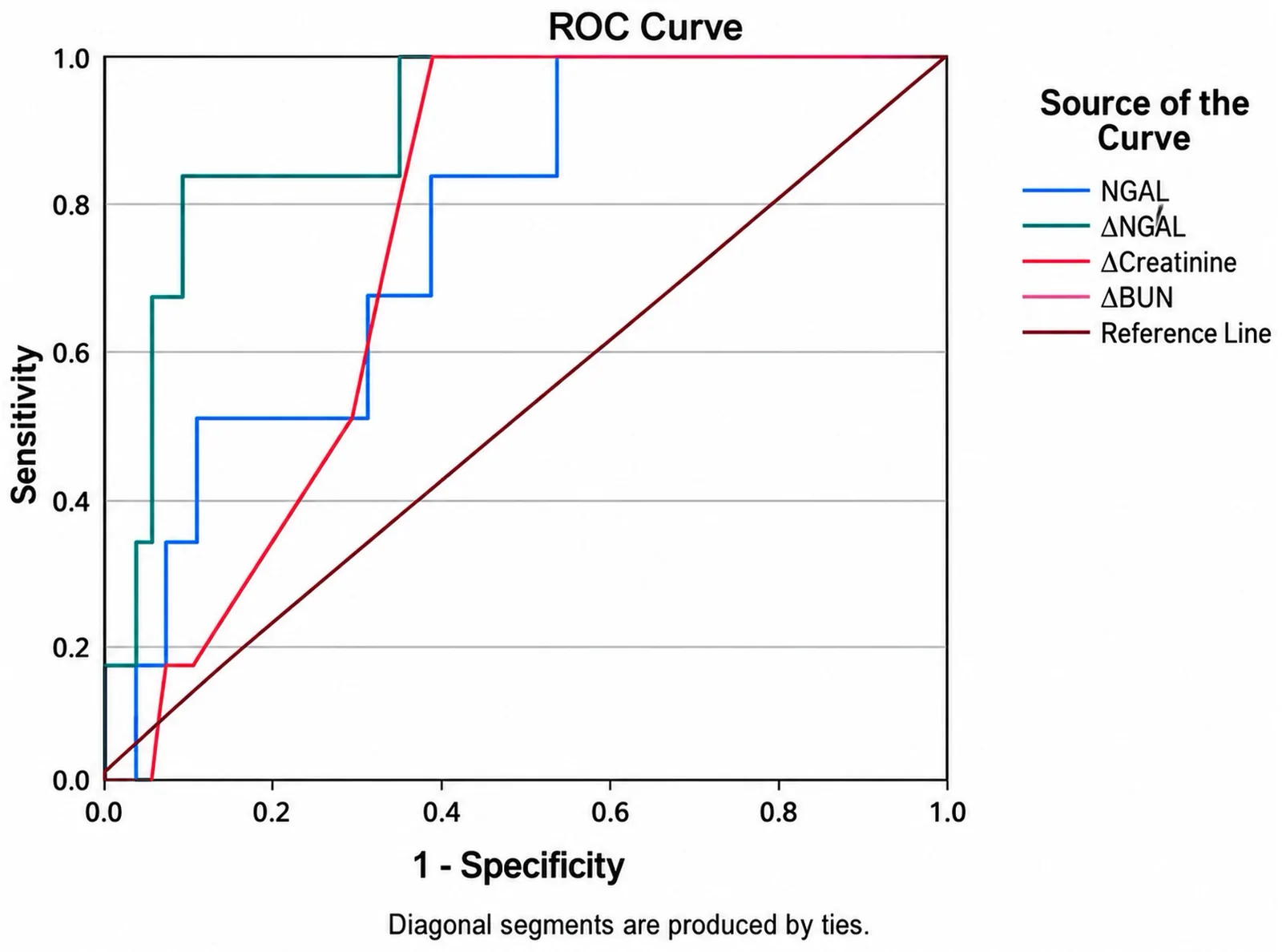
Receiver operating characteristic curves for the discrimination of postoperative acute kidney injury. ROC curves showing the diagnostic performance of day 1 preoperative changes in serum biomarkers for discriminating postoperative AKI after LSG. ΔNGAL demonstrated strong discriminatory performance for AKI discrimination, whereas Δcreatinine was interpreted as a reference criterion because AKI was defined using creatinine-based KDIGO criteria. BUN: blood urea nitrogen; NGAL: neutrophil gelatinase-associated lipocalin; ROC: receiver operating characteristic.

**Table 1:** Baseline characteristics and perioperative laboratory findings according to postoperative acute kidney injury status.

Variable	Time/Category	Total (*N* = 60)	AKI (*N* = 6)	Control (*N* = 54)	*p* Value
Age (years)		37.8 ± 11.4	39.3 ± 8.1	37.6 ± 11.7	0.725 ^α^
Sex	Male	10 (16.7%)	1 (16.7%)	9 (16.7%)	>0.999 ^β^
	Female	50 (83.3%)	5 (83.3%)	45 (83.3%)	
BMI (kg/m^2^)		44.2 (42–47.9)	42.6 (40.6–53.4)	44.5 (42.1–47.7)	0.767 ^θ^
ASA	I	37 (61.7%)	3 (50.0%)	34 (63.0%)	0.157 ^β^
	II	21 (35.0%)	2 (33.3%)	19 (35.2%)	
	III	2 (3.3%)	1 (16.7%)	1 (1.9%)	
NGAL (ng/mL)	Preoperative	99.8 (82.5–115.8)	87.7 (41–178.2)	100.4 (84.2–115.5)	0.430 ^θ^
	Day 1	123.3 (97.8–143.4)	148.6 (125.5–243.5)	121.8 (93.2–142.6)	0.041 ^θ^
	Day 3	143.7 (133.8–158.9)	140.7 (131–320.6)	143.9 (133.8–158.1)	0.892 ^θ^
Creatinine (mg/dL)	Preoperative	0.81 (0.78–0.9)	0.6 (0.6–0.7)	0.83 (0.8–0.91)	<0.001 ^θ^
	Day 1	0.8 (0.7–0.9)	0.95 (0.9–1)	0.79 (0.7–0.88)	0.004 ^θ^
	Day 3	0.76 ± 0.14	0.72 ± 0.1	0.77 ± 0.15	0.499 ^α^
BUN (mg/dL)	Preoperative	12 (10–14)	10.5 (8.8–12.5)	12 (10–15)	0.293 ^θ^
	Day 1	10 (8–11)	11 (9.2–11.5)	9 (8–11)	0.350 ^θ^
	Day 3	9 (8–10.8)	9 (8.2–10)	9.5 (7.8–11)	0.654 ^θ^
eGFR (mL/min/1.73 m^2^)	Preoperative	94.5 ± 16.4	113.7 ± 11.3	92.3 ± 15.6	0.002 ^α^
	Day 1	99.4 ± 18.7	81.8 ± 18.2	101.3 ± 17.9	0.014 ^α^
	Day 3	104.3 ± 15.2	109.2 ± 6.1	103.8 ± 15.8	0.417 ^α^
WBC (×10^9^/L)	Preoperative	9.6 ± 2.6	11.1 ± 4	9.4 ± 2.4	0.134 ^α^
	Day 1	11.55 (9.93–14.68)	11.3 (10.38–19.88)	11.75 (9.88–14.62)	0.640 ^θ^
	Day 3	9.4 ± 2.6	10.4 ± 4.5	9.3 ± 2.3	0.576 ^α^
Neutrophil count (×10^9^/L)	Preoperative	5.8 ± 1.8	6.1 ± 1.4	5.7 ± 1.9	0.617 ^α^
	Day 1	9.5 (7.53–12.89)	8.7 (7.97–14.19)	9.65 (7.46–12.7)	0.796 ^θ^
	Day 3	6 ± 2.1	6 ± 2.2	6 ± 2.1	0.958 ^α^
Lymphocyte count (×10^9^/L)	Preoperative	2.8 (2.2–3.4)	3.15 (2.12–6.47)	2.8 (2.2–3.4)	0.402 ^θ^
	Day 1	1.6 (1.1–2.15)	2.06 (1.43–3.25)	1.6 (1.09–2)	0.251 ^θ^
	Day 3	2.5 (1.97–3)	2.85 (1.27–5.69)	2.45 (1.99–2.92)	0.423 ^θ^
NLR	Preoperative	1.99 (1.7–2.48)	1.7 (1.16–2.52)	2 (1.7–2.52)	0.273 ^θ^
	Day 1	7.1 ± 4.3	5.5 ± 2.6	7.3 ± 4.5	0.335 ^α^
	Day 3	2.23 (1.73–3)	1.79 (1.36–3.05)	2.28 (1.8–3.02)	0.126 ^θ^
Hemoglobin (g/dL)	Preoperative	13.5 ± 1.5	12.5 ± 2.1	13.6 ± 1.4	0.075 ^α^
	Day 1	12.3 ± 1.3	11.5 ± 1.6	12.4 ± 1.3	0.115 ^α^
	Day 3	11.6 ± 1.7	11.1 ± 1.6	11.6 ± 1.7	0.463 ^α^
Glucose (mg/dL)	Preoperative	94.5 (82.2–113.8)	126.5 (90–169)	94.5 (82–113)	0.213 ^θ^
	Day 1	113.5 (95–135.5)	114 (97–134)	112.5 (95–138.2)	0.961 ^θ^
	Day 3	80 (70–99.8)	81 (74.5–102.8)	78.5 (70–99.2)	0.657 ^θ^
CRP (mg/L)	Preoperative	9.5 ± 5.2	10.2 ± 3.8	9.4 ± 5.3	0.706 ^α^
	Day 1	20.85 (13.22–29.43)	21.75 (13.57–65.47)	20.85 (13.03–29.27)	0.482 ^θ^
	Day 3	37.9 (23.75–51.12)	37.15 (32.5–80.17)	37.9 (21.57–51.48)	0.514 ^θ^
Albumin (g/L)	Preoperative	36.4 (35.4–40.23)	36.6 (35.23–37.95)	36.4 (35.25–40.4)	0.844 ^θ^
	Day 1	38.5 ± 3.1	37.7 ± 2.1	38.6 ± 3.2	0.503 ^α^
	Day 3	37.1 ± 3.5	36.7 ± 1.9	37.1 ± 3.6	0.780 ^α^
Total protein (g/L)	Preoperative	66.1 (64.12–68.35)	66.7 (59.67–68.5)	66.1 (64.17–68.12)	0.863 ^θ^
	Day 1	66.9 ± 4.8	67.3 ± 3.6	66.8 ± 5	0.812 ^α^
	Day 3	64.7 ± 5.1	66.8 ± 2.5	64.4 ± 5.2	0.291 ^α^
Operation duration (min)		73.7 ± 11.3	71.0 ± 12.4	73.9 ± 11.3	0.585 ^α^
Anesthesia duration (min)		96.6 ± 9.4	95.0 ± 10.0	96.7 ± 9.4	0.678 ^α^
Length of hospital stay (days)		4.1 ± 0.2	4.2 ± 0.4	4.0 ± 0.2	0.567 ^α^
Estimated blood loss (mL)		73.3 ± 25.2	60.0 ± 22.4	74.5 ± 25.2	0.294 ^α^
Intraoperative IV fluid administration (mL)		1029.0 ± 94.5	1000.0 ± 100.0	1031.8 ± 94.5	0.550 ^α^
Urine output (mL/h) *	Day 1	86.5 (75.2–103.5)	71 (61.5–81)	89.5 (76.8–106.5)	0.008 ^θ^
	Day 3	117.5 (107.2–135.5)	111.5 (107–126.8)	119.5 (106.8–136.5)	0.423 ^θ^

Data are presented as mean ± standard deviation, median (Q1–Q3), or n (%), according to distribution. ^α^ Independent-samples *t*-test (Welch corrected when required); ^β^ Fisher’s exact test; ^θ^ Mann–Whitney U test. AKI: acute kidney injury; ASA: American Society of Anesthesiologists; BMI: body mass index; BUN: blood urea nitrogen; CRP: C-reactive protein; eGFR: estimated glomerular filtration rate; NGAL: neutrophil gelatinase-associated lipocalin; NLR: neutrophil-to-lymphocyte ratio; WBC: white blood cell count. * Hourly urine volume was recorded in mL and normalized per kg for KDIGO assessments; Table 1 presents the mean 24 h volume in mL/h.

**Table 2:** Perioperative dynamic changes in serum NGAL and renal function parameters according to postoperative acute kidney injury status.

Variable	Time Interval	Total	AKI	Control	*p* Value
ΔNGAL (ng/mL)	Day 1–Preoperative	22.6 ± 35.3	72.7 ± 32.6	17.1 ± 31.1	<0.001 ^α^
	Day 3–Preoperative	54.8 (35.5–64.5)	83.3 (56.9–171.1)	52.3 (31.1–63.6)	0.014 ^θ^
	Day 3–Day 1	29.8 (3.6–55.5)	16.4 (−11.7–77.1)	33.1 (9.5–55.8)	0.349 ^θ^
ΔCreatinine (mg/dL)	Day 1–Preoperative	−0.1 (−0.1–0.02)	0.3 (0.3–0.33)	−0.1 (−0.13–−0.01)	<0.001 ^θ^
	Day 3–Preoperative	−0.1 (−0.17–0)	0.11 (−0.03–0.17)	−0.1 (−0.18–−0.01)	0.003 ^θ^
	Day 3–Day 1	−0.03 (−0.1–0.05)	−0.23 (−0.33–−0.14)	−0.01 (−0.09–0.06)	0.005 ^θ^
ΔBUN (mg/dL)	Day 1–Preoperative	−2 (−4.8–0)	−0.5 (−1–0.5)	−2.5 (−5–0)	0.044 ^θ^
	Day 3–Preoperative	−2.7 ± 3.2	−1.8 ± 2.3	−2.8 ± 3.3	0.478 ^α^
	Day 3–Day 1	0 (−2–1)	−1.5 (−4.2–0)	0 (−2–1)	0.210 ^θ^
ΔeGFR (mL/min/1.73 m^2^)	Day 1–Preoperative	10.5 (−3.8–15.8)	−34.5 (−37–−27.5)	11 (1.8–17.2)	<0.001 ^θ^
	Day 3–Preoperative	9.9 ± 12.8	−4.5 ± 10.1	11.5 ± 12.1	0.003 ^α^
	Day 3–Day 1	4 (−4–9.8)	29.5 (19.2–40)	0.5 (−4.2–8)	0.005 ^θ^

Data are presented as mean ± standard deviation or median (Q1–Q3), according to distribution. Δ: change between specified time points. ^α^ Independent-samples *t*-test; ^θ^ Mann–Whitney U test. AKI: acute kidney injury; BUN: blood urea nitrogen; eGFR: estimated glomerular filtration rate; NGAL: neutrophil gelatinase-associated lipocalin.

**Table 3:** Correlations between dynamic changes in serum NGAL levels and changes in renal function parameters.

Parameter	Time Interval	ΔNGAL Day 1–Preop r	*p*	ΔNGAL Day 3–Preop r	*p*	ΔNGAL Day 3–Day 1 r	*p*
ΔCreatinine	Day 1–Preoperative	0.297	0.021	0.113	0.388	−0.177	0.176
	Day 3–Preoperative	0.204	0.117	0.176	0.177	−0.050	0.703
	Day 3–Day 1	−0.165	0.206	−0.050	0.702	0.111	0.397
ΔBUN	Day 1–Preoperative	0.264	0.041	0.013	0.922	−0.151	0.248
	Day 3–Preoperative	0.148	0.259	0.032	0.807	−0.122	0.352
	Day 3–Day 1	−0.096	0.464	0.051	0.698	0.026	0.846
ΔeGFR	Day 1–Preoperative	−0.339	0.008	−0.191	0.143	0.122	0.353
	Day 3–Preoperative	−0.221	0.089	−0.129	0.324	0.077	0.560
	Day 3–Day 1	0.181	0.167	0.151	0.250	−0.061	0.642

Δ: change between specified time points; BUN: blood urea nitrogen; eGFR: estimated glomerular filtration rate; NGAL: neutrophil gelatinase-associated lipocalin.

**Table 4:** Diagnostic performance of day 1 preoperative biomarker changes for discriminating postoperative acute kidney injury.

Biomarker	AUC (95% CI)	*p* Value	Cut-Off	Sensitivity, % (95% CI)	Specificity, % (95% CI)
Δcreatinine	1.000 (1.000–1.000)	<0.001	0.3 mg/dL	100 (54.1–100)	100 (93.4–100)
ΔNGAL	0.901 (0.793–1.000)	0.001	57.2 ng/mL	83.3 (35.9–99.6)	90.7 (79.7–96.9)
NGAL Day 1	0.756 (0.523–0.989)	0.041	128.6 ng/mL	83.3 (35.9–99.6)	61.1 (46.9–74.1)
ΔBUN	0.750 (0.619–0.881)	0.046	−2.5 mg/dL	100 (54.1–100)	50.0 (36.1–63.9)

AUC: area under the curve; CI: confidence interval; Δ: change between specified time points; BUN: blood urea nitrogen; NGAL: neutrophil gelatinase-associated lipocalin. Sensitivity and specificity CIs are exact binomial 95% CIs. Cut-offs were selected using the Youden index and should be considered exploratory. Δcreatinine was included only as a reference because AKI was defined using creatinine-based KDIGO criteria.

## Data Availability

The datasets generated and/or analyzed during the current study are available from the corresponding author upon reasonable request, subject to institutional and ethical restrictions.
